# Investigating morphological awareness and the processing of transparent and opaque words in adults with low literacy skills and in skilled readers

**DOI:** 10.1111/1467-9817.12036

**Published:** 2014-08-25

**Authors:** Nancy L. To, Elizabeth L. Tighe, Katherine S. Binder

**Affiliations:** University of Massachusetts Amherst, Amherst, MA, USA; Florida State University, Tallahassee, FL, USA; Mount Holyoke College, South Hadley, MA, USA

## Abstract

For adults with low literacy skills, the role of phonology in reading has been fairly well researched, but less is known about the role of morphology in reading. We investigated the contribution of morphological awareness to word reading and reading comprehension and found that for adults with low literacy skills and skilled readers, morphological awareness explained unique variance in word reading and reading comprehension. In addition, we investigated the effects of orthographic and phonological opacity in morphological processing. Results indicated that adults with low literacy skills were more impaired than skilled readers on items containing phonological changes but were spared on items involving orthographic changes. These results are consistent with previous findings of adults with low literacy skills reliance on orthographic codes. Educational implications are discussed.

Adequate levels of language and literacy are necessary for adults to function in society, at work, and at home. The 2003 National Assessment of Adult Literacy collected data on basic (word-level) reading, prose literacy, document literacy, quantitative literacy, and health literacy skills from over 19,000 adults. The results indicated that approximately 40% of adults living in the United States, or 90 million adults, perform at or below the basic level of literacy required to function in daily life, which is approximately at the eighth-grade reading level (Kutner, Greenberg, & Baer, 2005). Adults with low levels of literacy reported higher rates of hospitalization, poverty, and involvement in the criminal justice system (Kutner et al., 2005). Given the negative implications that poor literacy skills have on an individual’s quality of life and for society at large, it is important to understand the factors that may contribute to adults’ language and literacy skills and to investigate cognitive processes related to adults’ level of literacy.

There is a small, but growing, body of literature that has begun to describe the relative strengths and weaknesses of adults with low literacy skills. This research documents the relative weakness in phonological awareness compared with a relative strength in orthographic awareness for these individuals (Greenberg, Ehri, & Perin, 1997, 2002; Thompkins & Binder, 2003). Although phonological and orthographic awareness are elements of metalinguistic awareness, morphological awareness is also an important component (Apel, Wilson-Fowler, Brimo, & Perrin, 2012; Deacon, 2012; Roman, Kirby, Parrila, Wade-Woolley, & Deacon, 2009). Morphological awareness is the conscious understanding of how words can be broken down into smaller units of meaning (Carlisle, 2000). Several researchers have recently argued that all three skills are necessary to promote growth in word learning and reading comprehension in children (Berninger, Abbott, Nagy, & Carlisle, 2010; Deacon, 2012; Kruk & Bergman, 2013; Tong, Deacon, Kirby, Cain, & Parrila, 2011). To date, only one other study has investigated the role of morphological awareness in reading comprehension for adults with low literacy skills (Tighe & Binder, 2014); however, that study did not examine the contribution of morphological awareness to reading words in isolation. The current study had two major aims. The first aim was to examine the relative contributions of pseudoword decoding and morphological awareness to word reading and reading comprehension. The second aim was to investigate the effects of word shifts (i.e., changes to the sound and/or spelling of the root) in morphological processing during a production-based task measuring accuracy and response times in a sample of adults with low literacy skills and skilled adult college readers.

## Morphological awareness

The English language does not rely exclusively on the alphabetic principle because letter-to-sound correspondences are not necessarily mapped one to one. Instead, English orthography is deep in the sense that a single sound can be represented by several graphemes (e.g., ‘c’ and ‘k’ can both produce the same sound). Therefore, the English writing system is better described as morphophonemic because words are spelled according to how they sound (phonemes) and what they mean (morphemes) (Chomsky & Halle, 1968; Venezky, 1970). For example, the word *hoped* is spelled with the suffix -*ed*, a past tense marker, rather than *hopt*. Because the ultimate goal of reading is comprehension, phonological and orthographic word forms must be mapped onto semantic information. The smallest phonological units that carry semantic information are morphemes, such as base words, prefixes, and suffixes. The ability to consciously manipulate morphemes and to employ word formation rules in one’s language is referred to as morphological awareness (Carlisle, 1995; Kuo & Anderson, 2006), whereas morphological processing refers to the implicit ability to understand and decipher morphologically complex words during reading and language comprehension.

In addition to roots, there are two main classes of morphemes. Inflectional morphemes are suffixes that preserve the stem but change its tense (*cook* to *cooked*) or quantity (*cat* to *cats*). Derivational morphemes, on the other hand, can change the meaning of a word (adding *ir*- to *regular* to form *irregular*), as well as change the part of speech (adding -*ly* to *slow* to form *slowly*). By the time they are 4 years old, children may demonstrate knowledge of inflectional morphology in oral language (e.g., past tense markers and pluralization; Anglin, 1993; Berko, 1958; Brown, 1973; Deacon & Kirby, 2004; Vogel, 1977) and emerging knowledge of derivational morphology (Carlisle, 1995, 2000; Tyler & Nagy, 1989). Conscious knowledge of the meanings of derivational morphemes (i.e., affixes that change the part of speech and/or meaning of the root) seems to develop in higher reading grade levels, as reflected by a drastic increase in morphological awareness between third and sixth grades (Anglin, 1993; Berninger et al., 2010) and shows a much longer developmental trajectory (Anglin, 1993; Berninger et al., 2010; Carlisle, 2000, 2003; Derwig & Baker, 1979; Freyd & Baron, 1982; Kruk & Bergman, 2013; Tong et al., 2011).

## Morphological awareness and reading

It is well established in the educational, psychological, and psycholinguistics research on children that morphological awareness plays an important role in word reading (Carlisle, 2000; Mahony, Singson, & Mann, 2000) and reading comprehension (Carlisle, 2000; Deacon & Kirby, 2004; Jarmulowicz, Hay, Taran, & Ethington, 2008; Nagy, Berninger, & Abbott, 2006), with findings of moderate correlations between morphological awareness and word reading and between morphological awareness and reading comprehension.

Phonological awareness has a greater impact on reading through the early elementary years, but morphological awareness plays an increasingly important role in reading through the later elementary years and beyond (Deacon & Kirby, 2004; Jarmulowicz et al., 2008; Nagy et al., 2006; Singson, Mahony, & Mann, 2000). In fact, Nagy and Anderson (1984) estimated that 60% of unfamiliar words encountered by school aged children are morphologically complex and can be understood by using familiar morphemes to infer their meanings. Morphological awareness aids word recognition and reading comprehension by breaking down words into small meaningful units (such as prefixes, base words, and suffixes), creating more word associations in the mental lexicon, and improving the ability to define unfamiliar words (Carlisle, 2000; Perdijk, Schreuder, & Verhoeven, 2005).

Although phonological awareness and morphological awareness are related, research also shows that each provides a unique contribution to word reading and reading comprehension in upper elementary school children (Carlisle, 2000; Singson et al., 2000). Several studies have found that morphological awareness explains variance in real and pseudoword decoding abilities (Deacon, 2012; Deacon & Kirby, 2004; Roman et al., 2009; Singson et al., 2000). Singson and colleagues (2000) noted that above and beyond the 16% contribution of short-term verbal memory, morphological awareness provided a small (5%), but significant and unique contribution to explaining the reading variance among students in grades 3 through 6. Furthermore, in a series of hierarchical regressions for each grade level in which morphological awareness was entered first, the contribution of phonological awareness failed to reach significance in explaining total reading variance beyond the third grade. Their research suggested that morphological awareness played an increasingly important role beginning in fourth grade and provided a significant contribution, above and beyond phonological awareness, to reading ability in fifth and sixth grades.

Nagy et al. (2006) investigated the contributions of morphological awareness, phonological working memory, and phonological decoding to reading comprehension, reading vocabulary, and spelling among fourth/fifth, sixth/seventh, and eighth/ninth graders. For the two lower level reading groups (grades 4 through 7), only morphological awareness emerged as a significant unique predictor of reading comprehension. In contrast, for eighth/ninth graders, all three skills (phonological working memory, phonological decoding, and morphological awareness) accounted for unique variance in reading comprehension. For eighth/ninth graders, morphological awareness accounted for a greater percentage of the variance in reading comprehension than phonological working memory and decoding.

Tong et al. (2011) demonstrated a relationship between reading comprehension abilities and the rate at which children learn derivational morphology, showing that stronger reading comprehension abilities are associated with early mastery of morphological abilities. Children with poor or average reading comprehension ability in third grade performed worse than third graders with good comprehension abilities in derivational morphology despite adequate phonological awareness, orthographic, decoding, and naming speed skills. Thus, the first goal of this investigation was to examine the role of morphological awareness to the word reading and reading comprehension abilities of adults with low literacy skills and skilled college readers, after controlling for decoding abilities.

## Morphological complexity and opacity

One characteristic of morphemes that obscures the internal structure of a word and the semantic relationship between base and derived form is referred to as opacity, which can take phonological or orthographic forms (Carlisle, 2000). Phonological opacity, or a phonological change, occurs when a suffix is added to a base word and there is an alteration in the stress and/or vowel sound to create the derived word form. Orthographic opacity, or an orthographic change, occurs when the addition of a suffix alters the spelling of the base word to create the derived word form. Carlisle (1987, 1988) and Leong (1999) categorized relationships between a base word form and its derived word form into four types: (1) no change (e.g., *dry–dryer*), (2) orthographic change (e.g., *begin–beginner*), (3) phonological change (e.g., *courage–courageous*), and (4) both orthographic and phonological change (e.g., *deep–depth*). Opacity affects the accuracy and speed of producing and reading morphologically complex words (Carlisle, 1988, 2000; Leong, 1999).

Researchers consistently find that students are most likely to orally produce derived forms accurately when no change in the orthographic or phonological form is required (Carlisle, 1987, 1988; Clin, Wade-Woolley, & Heggie, 2009; Fowler & Liberman, 1995; Jarmulowicz, 2006). The ability to identify a base form given a morphologically complex derived form when there is a phonological or both change (in which there is some phonological complexity) distinguished less skilled and skilled readers in upper elementary school children (Fowler & Liberman, 1995). The ability to read morphologically complex words, especially derived words, may be complicated by the fact that they are typically longer (multisyllabic words), lower in frequency, more abstract in meaning, and more phonologically and orthographically complex.

Carlisle (1988) investigated morphological awareness in fourth, sixth, and eighth graders. Students were asked to provide derivations in response to base words and to name base words of derived forms. In the derivation task, students were given the example, ‘Warm. He chose the jacket for its (warmth)’, and an example of decomposition would be supplying the word *danger* in a sentence after hearing *dangerous*. The students were more successful in providing the base words when given the derived form than providing derived words when given the base form, and older students were more successful than younger students. The most difficult word conversions involved phonological changes, such as *admit/admission*. Middle-school students demonstrated weaker oral production performance when producing derived forms of words with both orthographic and phonological changes (e.g., *decide* to *decision*) than on words transparent in both phonology and orthography (e.g., *enjoy* to *enjoyment*), phonological changes (e.g., *magic* to *magician*), and orthographic changes (e.g., *day* to *daily*).

Leong (1999) examined processing differences across word shift types for college students with learning disabilities compared with skill and age-matched participants. In addition to examining accuracy, as was carried out in previous work, he importantly examined response times to the target items and found that participants took longer to respond to items that included a phonological shift (i.e., phonological shift and both shift items) compared with the stable and orthographic shift items. Thus, even college students display a sensitivity to phonological shifts in morphologically complex words.

## Current study

To date, only one other study has investigated the relative contribution of decoding and morphological awareness to reading comprehension in adults with low literacy skills (Tighe & Binder, 2014), and only one other study has investigated the effect of phonological and/or orthographic opacity and transparency in adult readers (Leong, 1999). Therefore, the first goal of the present study was to investigate the contribution of morphological awareness, after controlling for pseudoword decoding, to word reading, as this variable was not examined in the prior study, and reading comprehension in adults with low literacy skills and skilled, college students. Our second goal was to investigate the effects of word change conditions (stable, phonological, orthographic, and both change) on the accuracy and response times of morphological production in adults with low literacy skills and skilled college readers to examine if the effects Leong found extend to a population of adults with low literacy skills.

To address the first research question, we used hierarchical regression analyses. We hypothesized that morphological awareness would consistently provide a unique contribution to both the word reading and comprehension abilities of adults with low literacy skills and skilled college readers. To address the second research question, we investigated the effect of word changes (stable, phonological, orthographic, both change) on accuracy and response times for both reader groups. To accomplish this, we utilized a morphological oral production task (similar to Carlisle, 1988), in which participants provided the derived form of a given base word to complete a sentence. We expected adults with low literacy skills to respond differentially to items that involved an orthographic or phonological change than skilled adult readers. Although we believed that all participants would be less accurate and take longer to respond to items that involved a phonological change, we hypothesized that the cost associated with these items would be smaller for skilled readers because they have better phonological awareness and better phonological processing skills compared to adults with low literacy skills (e.g., Bell & Perfetti, 1994; Binder & Borecki, 2007). In addition, we hypothesized that items containing orthographic changes would be easier compared with the phonological change items for our adults with low literacy skills because they have demonstrated a relative strength with this information (Greenberg et al., 1997, 2002; Thompkins & Binder, 2003).

## Method

### Participants

Our participants included 61 adults with low literacy skills and 89 college students. The adults with low literacy skills were recruited from two Adult Basic Education (ABE) centers in Massachusetts. These ABE centers provide coursework and instruction for adults to earn a high school equivalency diploma (i.e., General Educational Development [GED] certificate). The adults with low literacy skills consisted of 73% women and 27% men, and the participants ranged in age from 17 to 60 years (*M* = 27.6). Their reading grade levels, using the Word Attack subtest from the Woodcock–Johnson Achievement test, ranged from 2.4 to 18 (*M* = 7.09). Among the adults with low literacy skills, the average highest grade level completed was the ninth grade. Approximately 9.1% of these participants self-reported a diagnosed learning disability. Demographic information was unavailable for six of the adults with low literacy skills.

The college students were recruited from a private, women’s liberal arts college in Massachusetts through flyers, emails, and word of mouth. All of the college students in this sample were female. Their ages ranged from 18 to 44 years (*M* = 20), and their reading grade level, using the Word Attack subtest from the Woodcock–Johnson Achievement test, ranged from 4.7 to 18 (*M* = 12.54). None of the college students received specialized reading instruction in grade school. On a standardized reading comprehension assessment (i.e., Nelson Denny Reading Comprehension test), all college participants responded with at least 70% accuracy. Thus, the college students in this sample represent a skilled-reader group. See Table 1 for the means and group differences across the achievement measures for our two reader groups.

The proportions of non-native English speakers and native English speakers in the two reading groups were similar. Approximately 65% of the adults with low literacy skills were native English speakers, and 28% of the adults with low literacy skills listed another language as their native language (26.2% Spanish and 1.6% Portuguese). Our participant demographics are consistent with those reported from ABE programmes across the United States, consequently making our sample representative of this group (Lesgold & Welch-Ross, 2012).

### Materials

#### Pseudoword decoding: Woodcock–Johnson Psycho-Educational Battery III – Word Attack Subtest (Woodcock, McGrew, & Mather, 2001)

The Word Attack subtest from the Woodcock–Johnson is a norm-referenced measure of pseudoword decoding. Each participant was asked to pronounce a list of nonwords of increasing difficulty, such as *nat*, *feap*, and *mibgus*. Standardized procedures require testing to be discontinued after six consecutive errors. Participants’ scores were the total number of correctly read letters and words. The median reliability for the Word Attack subtest for adults is .87 (Woodcock et al., 2001). Binder, Snyder, Ardoin, and Morris (2011) used this test in an ABE setting and obtained a Cronbach’s alpha of .94.

#### Word reading: Woodcock–Johnson Psycho-Educational Battery III – Letter-Word Identification (Woodcock, McGrew, & Mather, 2001)

The Letter-Word Identification subtest required participants to read aloud real words, presented in isolation and in order of increasing difficulty. Administration was terminated when participants made six consecutive errors. The median reliability for the Letter-Word Identification subtest was .94 for adults (Woodcock et al., 2001). Binder et al. (2011) used this test in an ABE setting and obtained a Cronbach’s alpha of .95.

#### Reading comprehension: Woodcock–Johnson Psycho-Educational Battery III – Passage Comprehension (Woodcock, McGrew, & Mather, 2001)

The Passage Comprehension subtest is a measure of reading comprehension, using a cloze sentence procedure. Items were presented in an order of increasing difficulty, and administration was discontinued when six consecutive items were missed. The median reliability for the Passage Comprehension subtest for adults was .88 (Woodcock et al., 2001). Binder et al. (2011) reported a Cronbach’s alpha of .92 with ABE students.

#### Morphological awareness: base (BMorph) and derived (DMorph) form morphology production tasks (adapted from Carlisle, 2000)

For the production tasks (BMorph and DMorph), participants heard a single word followed by a sentence that contained a missing word. To accurately fill in the missing word, participants were required to orally provide the correct form of the first word given. For the BMorph task, the participant heard a morphologically complex word, followed by a sentence prompting the participant to decompose the derived word form to provide the base form to correctly complete the sentence. For example, ‘Driver. Children are too young to (drive)’. (see Appendix A). For the DMorph task, a base form of a word was given, followed by a sentence designed to elicit a derived form of the word. For example, ‘Farm. My uncle is a (farmer)’. The purpose of this measure was to assess explicit knowledge of derivational morphemes. In both tasks, eight items included a phonological change, eight items contained an orthographic change, eight items were both change, and eight items were no change (also called stable). BMorph and DMorph each consisted of 32 total items. The targets for each of the change conditions were equated on word frequency, on the basis of Francis and Kucera norms (1982), for both the root of the word and the derived form. That is, there were no significant differences among the means for the root or the derived form for either BMorph, *F*(3,28)=0.11, *MSE* = 1610.63, *p* = .96, *F*(3,28) = 0.10, *MSE* = 2235.92, *p* = .96, respectively, or DMorph, *F*(3,28)=0.01, *MSE* = 4572.15, *p* = .99, *F*(3,28)=0.34, *MSE* = 1841.31, *p* = .80, respectively. See Table 2 for the root and derived form means and standard deviations for each of the four BMorph and DMorph conditions.

The sentences for the subtests were prerecorded onto audio files by a female speaker and programmed into E-PRIME to play in a randomized order. Three practice items were given before each task. The trial began by providing the target word in either its base or derived form, followed by the sentence that needed to be completed with a form of the provided word. The participant was then prompted to supply the target word. Once the computer detected the vocalization of the target word, the trial was over, and a fixation cross appeared until the participant pressed a key to move on to the next trial. Thus, response times reflect the amount of time it took the participants to provide a correct form of the initial word given, or a processing time. Response times were collected using E-PRIME, and accuracy scores were hand-recorded by the researcher. Any self-corrections or false starts were noted by the examiner and were coded as correct for the accuracy scores but were re-coded as a missing data point for the response time.

The accuracy scores of both BMorph and DMorph were combined to establish a composite morphological awareness score, as these variables were highly correlated (*r* = .64, *p* <.001). However, the accuracy and response times from DMorph were used to investigate participants’ processing of morphologically complex words.

#### Demographics questionnaire

A demographics questionnaire was given, asking participants to describe important aspects related to their language development and history, such as their native language, home country, number of years in the United States, age, ethnicity, gender, and the nature of a disability if the individual self-reported one.

### Procedure

Participants were given one testing session, lasting 40 to 50 minutes. The adults with low literacy skills received $10 at the end of the testing session as compensation for participating in the study. College students received one research credit, which fulfilled a class research participation requirement for a class. The three Woodcock–Johnson tasks were always administered in the same order (word attack, letter-word identification, and passage comprehension), but this battery was counterbalanced with the two morphology tasks. In addition, we counterbalanced the order of the morphology tasks.

## Results

### Regression analyses using decoding and morphological awareness to explain variance in word reading and reading comprehension

Morphological awareness (average percent accuracy on the DMorph and BMorph tasks) was positively correlated with decoding, word reading, and passage comprehension (Table 3).

We conducted hierarchical regression analyses to investigate the contribution of morphological awareness to word reading, after pseudoword decoding skills were accounted for. These regression analyses were conducted for each reading group. For the adults with low literacy skills, decoding ability explained a significant amount of variance in the word reading, *F*(1,59)=37.5, *p* <.001, accounting for 38.9% of the variance. When morphological awareness was added in the second step, the increment in *R*^2^ was significant, *F*(1,58) =5.44, *p* <.05 (change in *R*^2^ = .05). Both pseudoword decoding and morphological awareness were unique predictors. For the skilled readers, decoding ability explained a significant amount of variance (22.6%) in word reading, *F*(1,87)=25.44, *p* <.001. There was a significant increase in the amount of variance explained (6.4%) in the second step for this group, *F*(1,86)=7.79, *p* <.01. Examination of the β weights shows that both variables were unique predictors of word reading (Table 4).

In our second set of regression analyses, we investigated the contribution of morphological awareness to reading comprehension, after pseudoword decoding skills were accounted for. For the adults with low literacy skills, decoding ability explained a significant amount of variance (11%) in the reading comprehension scores, *F*(1,59)=7.30, *p* <.01. When morphological awareness was added in the second step, the increment in *R*^2^ was significant (an additional 7%), *F*(1,58)=4.61, *p* <.05. When morphological awareness was added, decoding was not a unique predictor of reading comprehension but morphological awareness was. For the skilled readers, decoding ability explained a significant amount of variance (8%) in reading comprehension, *F*(1,87)=7.22, *p* <.01. There was a significant increase in the amount of variance explained (19%) in the second step for this group, *F*(1,86)=22.25, *p* <.001. Examination of the β weights shows that whereas decoding was not a unique predictor, morphological awareness was a unique predictor of reading comprehension for skilled readers (Table 5).

### ANOVAs on accuracy and response times to DMorph

Our second question investigated how adults with low literacy skills and skilled college readers perform on morphological production tasks, specifically on a morphological awareness measure that required participants to generate a morphologically complex word given the base form to complete a sentence across different word change conditions.

A 2 (Group: Low Literacy vs. Skilled Readers) ×4 (Word Change Condition: stable, phonological change, orthographic change, and both change) mixed methods ANOVA was used to determine if reading group and word change condition had a significant effect on the accuracy and response times on the DMorph task. Significant effects were followed up using Bonferroni post-hoc corrections. Effect sizes can be interpreted according to values of partial eta squared that correspond to Cohen’s (1988) benchmark *f* values for small, medium, and large effects (i.e., $\eta_{p}^{2}=.01$, .06, and .14, respectively).

**Figure 1:**
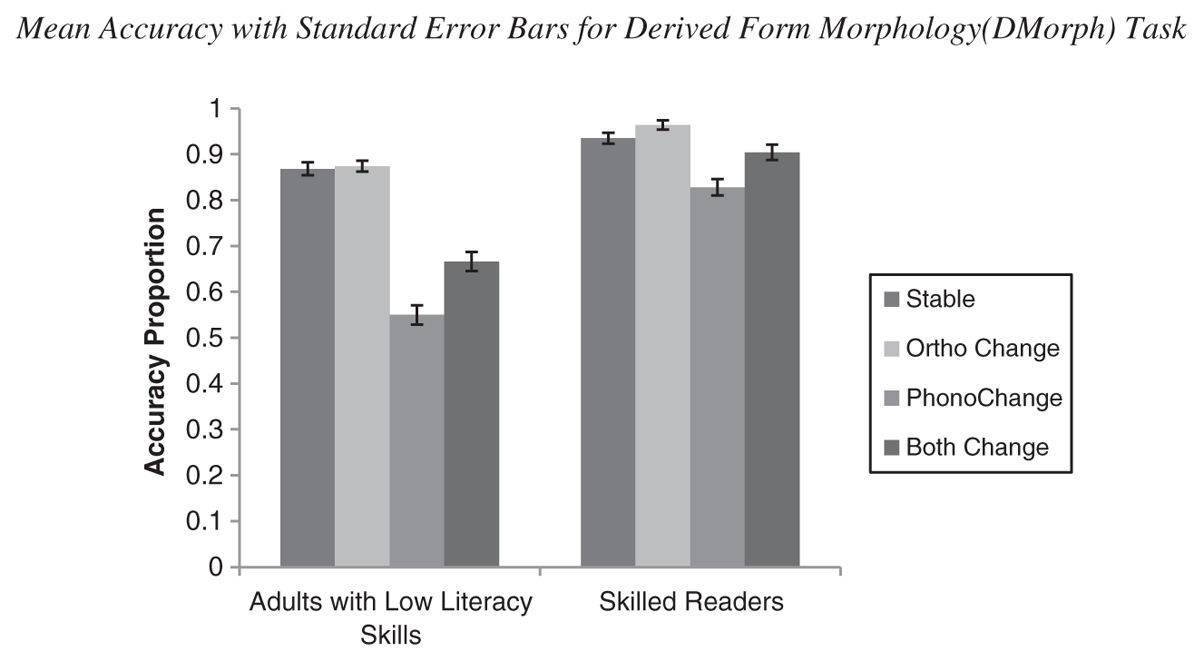
Mean accuracy with standard error bars for derived form morphology (DMorph) task.

Percent correct for production accuracy was used as the dependent variable in this analysis. See Figure 1 for means and standard errors. The main effect for reading group was significant, such that skilled readers (*M* = 90.8%, *SD* = 9.43) were significantly more accurate compared with adults with low literacy skills (*M* = 74.0%, *SD* = 9.37), *F*(1,148) =122.34, *MSE* = 0.034, *p* <.001, $\eta_{p}^{2}=.45$. In addition, performance across word change conditions differed significantly, such that participants were significantly less accurate on phonological change items (*M* = 68. 9%, *SD* = 21.52) compared with both change items (*M* = 78.5%, *SD* = 20.08), no change (*M* = 90.2%, *SD* = 11.56), and orthographic change (*M* = 91.9%, *SD* = 10.31) items, *F*(3,444)=120.15, *MSE* = 0.014, *p* <.001, $\eta_{p}^{2}=.45$.

The interaction between reading group and change type was also significant, *F*(3,444)=28.42, *MSE* = 0.014, *p* <.001, $\eta_{p}^{2}=.16$. For adults with low literacy skills, accuracy was highest for stable (*M* = 86.76%, *SD* = 13.67) and orthographic change (*M* = 87.4%, *SD* = 11.87) items, *p* >.05, but performance suffered significantly when a phonological change was present: phonological change items (*M* = 55.0%, *SD* = 20.52) and both change (*M* = 66.6%, *SD* = 18.01), *p*s <.001. Similarly, skilled readers were most accurate on the stable (*M* = 93.5%, *SD* = 8.93) and orthographic change items (*M* = 96.4%, *SD* = 7.10), and they were significantly less accurate on phonological change items (*M* = 82.8%, *SD* = 13.46) compared with all conditions, *p*s <.001. Although the both change items (*M* = 90.4%, *SD* = 15.10) were responded to less accurately compared with the orthographic and the phonological change items, *p*s <.001, there was no significant difference between the both change and the stable items, *p* = .45.

**Figure 2:**
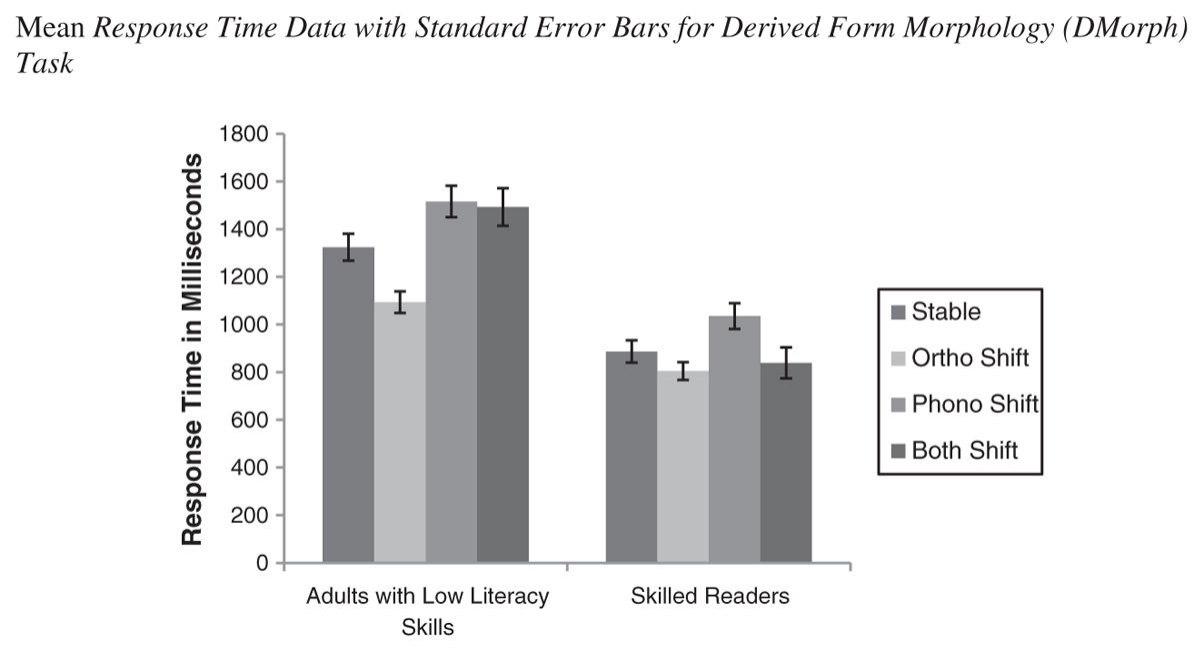
Mean response time data with standard error bars for derived form morphology (DMorph) task.

We used response times from the DMorph task to investigate the effect of word change conditions on morphological processing. The response times were screened four times (as practiced by Deacon, Parrila, & Kirby, 2006; Leong, 1999): (1) response times of incorrectly answered items were removed from the data set; (2) response times of skipped items were deleted (2.04%); (3) response times below 200 milliseconds were deleted (1.44%); (4) response times that were outliers, defined as two standard deviations (calculated separately for each reading group) from the mean (of each reading group), were removed (2.25%). See Figure 2 for means and standard errors.

We performed a 2 (Group) ×4 (Word Change Condition) mixed methods ANOVA. As expected, skilled readers (*M* = 891.23 ms, *SD* = 269.007) responded significantly faster than adults with low literacy skills (*M* = 1356.44 ms, *SD* = 466.48), *F*(1,148)=59.7, *MSE* = 525064, *p* <.001, $\eta_{p}^{2}=.29$. Performance on word change conditions also differed significantly, *F*(3,444)=18.62, *MSE* = 144228, *p* <.001, $\eta_{p}^{2}=.11$, such that orthographic change items (*M* = 949.05 ms, *SD* = 356.73) were responded to significantly faster than stable (*M* = 1105.05 ms, *SD* = 452.67), phonological (*M* = 1275.39 ms, *SD* = 522.53), or both change items (*M* = 1165.84 ms, *SD* = 622.46). Furthermore, items containing phonological change were responded to significantly slower than stable items, *p* <.001.

The interaction between reading group and word change condition using the response time data was also statistically significant, *F*(3,444)=5.66, *MSE* = 144228.45, *p* <.001, $\eta_{p}^{2}=.04$. Adults with low literacy skills responded to orthographic change (*M* = 1093.68 ms, *SD* = 424.99) significantly faster than stable (*M* = 1323.82 ms, *SD* = 586.37), phonological change (*M* = 1515.38 ms, *SD* = 678.08), and both change items (*M* = 1492.88 ms, *SD* = 887.43), *p*s <.05. No other differences were significant, *p*s >.05. Skilled readers responded significantly faster to orthographic change items (*M* = 804.41 ms, *SD* = 288.83) than stable (*M* = 886.29 ms, *SD* = 312.49), and phonological change items (*M* = 1035.41 ms, *SD* = 360.13), *p*s <.01. Skilled readers responded significantly faster on items containing both changes (*M* = 838.80 ms, *SD* = 303.27) than phonological changes, *p* <.001.

## Discussion

The purpose of this study was twofold: to investigate the contribution of morphological awareness to word reading and reading comprehension and to examine the effects of opacity in accuracy and response times. These questions were answered by examining the performance of adults with low literacy skills compared with skilled adult readers because much of the research investigating the relationships between decoding, morphological awareness, word reading, and reading comprehension has examined the developmental learning trajectories with samples of children. To answer the first set of questions, for each skill level, both decoding and morphological awareness were unique predictors of word reading, but only morphological awareness emerged as a unique predictor of reading comprehension for both groups. This finding suggests that within each group of readers, morphological awareness distinguishes between skilled and less skilled reading comprehension abilities. That is, even in the skilled reader group, morphological awareness accounted for significant variance, despite a range of comprehension abilities. The results of the opacity question were compelling as well. Both groups of readers demonstrated a relative strength in orthographic skills; accuracy and response times for items that contained an orthographic change were comparable to stable items (i.e., items that did not contain a change). However, they were less accurate and slower to respond to items that contained a phonological change. The morphological awareness abilities of adults with low literacy skills appear to be more impaired.

Consistent with past research (Tighe & Binder, 2014), we have demonstrated that morphological awareness significantly increases the proportion of variance explained in word reading and reading comprehension abilities, above and beyond decoding abilities (or knowledge of alphabetic principle/phonics) alone. This was true of the entire sample, and for each of the skill level groups.

When participants were asked to provide a morphologically complex word given the base form, both adults with low literacy skills and skilled readers were significantly less accurate on items containing phonological changes than all other word change conditions (stable, orthographic, and both). Overall, the accuracy and response time data for skilled readers, when asked to produce a morphologically complex word from a base word, indicated a reliance on phonological codes, such that when there was a disruption caused by a phonological change between the base and derived form, skilled readers were less accurate and had slower response times. This was true for the adults with low literacy skills, too, but their morphological skills were more impaired when the phonological structure of the base and derived form was opaque, which resulted in accuracy scores of 55% for the phonological change condition. Of course, this reliance on phonological codes could be due to the nature of the task; we were asking participants to produce a morphologically complex word. However, we suspect that the difficulty evident with items that have a phonological change would surface in other tasks that might require a different type of response. For example, if these words were embedded in a sentence context and participants’ eye movements were recorded, we would expect to see reading time differences on the phonologically opaque words compared with the stable words.

Our findings are consistent with past research (e.g., Fowler & Liberman, 1995; Leong, 1999) on processing of stable and opaque words. For example, Fowler and Liberman (1995) investigated stable and phonologically complex items in 7- and 9-year-olds. They found that identifying the base from a morphologically complex word when the base and derived form underwent a phonological change distinguished less skilled and skilled readers. Similarly, Leong (1999) found a general slowing in the phonological and both change conditions in college students and college students with reading disabilities.

Less skilled readers were more accurate on items with no phonological change (i.e., stable and orthographic change) than on items with a phonological change. Given their relative strength for remembering orthographic units (Greenberg, Ehri, & Perin, 1997, 2002; Thompkins & Binder, 2003), and that phonological and morphological awareness seem to provide contributions to their word reading and comprehension abilities, it seems reasonable that explicit morphological instruction may aid adults with low literacy skills’ vocabulary and comprehension. Unlike phonemes, morphemes provide information about syntax, meaning, spelling, and pronunciation.

### Implications for practice

Because the discrepancy in vocabulary knowledge by the time children enter school may already be quite large for children from disadvantaged socioeconomic or linguistic minority backgrounds compared with children from native English speaking households, it is essential for students to acquire strong skills for acquiring word meanings. Because many English words are morphologically related, learning one base word might increase the total vocabulary by a count of several words, if the student learns word formation processes of English. For example, if a person learns the word ‘love’, then morphologically related words (i.e., loveable, lovely) can also be acquired. It is also important to learn that affixes have regularity. For instance, learning that ‘-ment’ can be added to a root verb to create a noun version (e.g., establishment, employment) is useful. Learning that affixes can change the part of speech in consistent ways but can also alter the meaning is important as well. If a person knows the meaning of the word ‘happy’, and that the affix ‘un’ means the opposite, then he or she can ascertain the meaning of an unfamiliar word (e.g., unhappy). Although a word’s meaning may not be completely understood from one encounter, students can approximate a word meaning by parsing a complex word into familiar constituents. Thus, recognizing and understanding morphemes may simplify the task of word learning.

Learning word formation processes is also important for students who are non-native English speakers. This is especially true of ABE programmes in the United States in which the majority (62%) of non-native English speaking students are native Spanish speakers (Tamassia, Lennon, Yamamoto, & Kirsch, 2007). Research supports the importance of transfer to second language acquisition (oral and reading), particularly in Spanish and English to acquire novel vocabulary words in a second language (e.g., August, Carlo, Dressler, & Snow, 2005). For example, in Spanish, cognates may be taught to help students acquire English, such as *vacation* and *vacaciones*. The English suffix-*tion* is similar to the Spanish suffix function of the -*cion*. The English as a Second Language adult students may benefit from instruction that will connect phonological and orthographic units with morphological features. Therefore, explicit morphological instruction may be valuable to students, particularly around the time they begin to encounter more morphologically complex words in their reading materials.

A recent meta-analysis showed that morphological instruction was particularly effective for children with reading, learning, or speech and language disabilities, English language learners, and struggling readers (Goodwin & Ahn, 2010). Therefore, morphological instruction may remediate phonological processing difficulties, as children show gains in phonological awareness after morphological instruction. Thus, the findings suggest that students with literacy difficulties, such as adults with low literacy, may benefit from explicit morphological instruction.

The present study contributes to the current body of literature on adult literacy by systematically investigating morphological processing and the contribution of morphological awareness to various literacy outcomes over and above pseudoword decoding skills. In particular, we found that orthographic transparency may provide a greater benefit for adults with low levels of literacy in processing morphologically complex words, but phonological opacity poses a serious challenge to the processing of morphologically complex words among adults with low levels of literacy. These findings are consistent with other researchers who have investigated morphological awareness in college students with learning/reading disabilities (Leong, 1999), suggesting that both groups of adult readers (i.e., adults with low literacy skills from the current study and college students with learning/reading disabilities from the Leong, 1999 study) may benefit from a literacy curriculum that explicitly teaches morphological (word formation) rules and grammar.

## Figures and Tables

**Table 1:** Differences in achievement measures between adults with low literacy skills (*N* = 61) and skilled (*N* = 89) readers

Measure	Adults with low literacy		Skilled readers		Group differences	*p*
	*M*	*SD*	*M*	*SD*	*F*	
Word attack	24.00	4.01	28.62	2.32	79.38	<.001
Letter-word identification	62.66	4.64	71.30	3.04	189.97	<.001
Passage comprehension	33.21	3.46	38.83	3.51	93.90	<.001
Morphological awareness	81.78	8.67	92.70	5.26	91.90	<.001

*Note: SD*, standard deviation.

**Table 2:** Means and standard deviations of BMorph and DMorph conditions

Word condition	BMorph	DMorph
	*M* (*SD*)	*M* (*SD*)
Phonological change		
Root word form	37.38 (37.96)	60.75 (84.61)
Derived word form	53.88 (37.84)	47.63 (31.89)
Orthographic change		
Root form	43.88 (24.26)	60.50 (36.42)
Derived word form	53.50 (55.77)	35.25 (28.81)
Both change		
Root word form	48.50 (43.49)	60.75 (64.98)
Derived word form	61.88 (55.85)	52.38 (58.44)
Stable		
Root word form	45.13 (50.22)	60.75 (74.71)
Derived word form	63.50 (60.75)	55.25 (45.85)

*Notes:* BMorph, base form morphology; DMorph, derived form morphology; *SD*, standard deviation.

**Table 3:** Correlation coefficients between morphological awareness and decoding, word reading, and reading comprehension

Tasks	WJ	WJ	WJ

	Word Attack	Letter-Word ID	Passage Comp
Adults with low literacy skills			
Morphological awareness	.53***	.52***	.44***
Skilled readers			
Morphological awareness	.23*	.36**	.49***

*Notes*: WJ, Woodcock–Johnson Psycho-Educational Battery III.

*N* (adults with low literacy skills) = 61; *N* (skilled) = 89.

* *p* <.05;

** *p* <.01;

*** *p* <.001.

**Table 4:** Regression analysis of decoding and morphological awareness on word reading

Group and tasks	Δ in *R*^2^	β	*SE*	*t*
Adults with low literacy skills (*R*^2^ full model = .44)				
Decoding	.39***	.48	.13	4.17***
Morphological awareness	.05*	.27	6.18	2.33*
Skilled readers (*R*^2^ full model = .29)				
Decoding	.23***	.41	.12	4.41***
Morphological awareness	.06**	.26	5.40	2.79**

*Notes: SE*, standard error.

*N* (adults with low literacy skills) = 61; *N* (skilled) = 89.

* *p* <.05;

** *p* <.01;

*** *p* <.001.

**Table 5:** Regression analysis of decoding and morphological awareness on reading comprehension

Group and tasks	Δ in *R*^2^	β	*SE*	*t*
Adults with low literacy skills (*R*^2^ full model = .18)				
Decoding	.11	.17	.12	1.21
Morphological awareness	.07*	.30	5.56	2.15*
Skilled readers (*R*^2^ full model = .27)				
Decoding	.08	.17	.14	1.82
Morphological awareness	.19***	.45	6.33	4.72***

*Notes: N* (adults with low literacy skills) = 61; *N* (skilled) = 89.

SE, standard error.

* *p* <.05;

*** *p* <.001.

## Appendix A. Derived Form Morphology (DMorph) Task (adapted from Carlisle, 2000)

### Practice

- Farm. My uncle is a _____. [Farmer]
- Long. They measured the ladder’s _____. [Length]
- Human. The kind man was known for his _____. [Humanity]

### No-Shift Condition

1. Warm. He chose the jacket for its _____. [Warmth]
2. Differ. Their support makes a _____. [Difference]
3. Humor. The story was quite _____. [Humorous]
4. Remark. The speed of the car was _____. [Remarkable]
5. Reason. Her concerns were quite _____. [Reasonable]
6. Dry. Put the laundry in the _____. [Dryer]
7. Employ. It is difficult to find _____. [Employment]
8. Assist. The teacher will give you _____. [Assistance]

### Orthographic Shift Condition

1. Nine. The cyclist came in _____. [Ninth]
2. Glory. The view from the hilltop was _____. [Glorious]
3. Happy. Money does not buy _______. [Happiness]
4. Expense. The new car was _______. [Expensive]
5. Judge. He should have used better _____. [Judgment]
6. Secure. An alarm system provides ______. [Security]
7. Rely. The babysitter was very _______. [Reliable]
8. Begin. She was only a _____. [Beginner]

### Phonological Shift Condition

1. Confide. The girl was full of _____. [Confidence]
2. Equal. Boys and girls are treated with _____. [Equality]
3. Major. He won the vote by a _____. [Majority]
4. Drama. The actress was very _____. [Dramatic]
5. Engine. He works as an _____. [Engineer]
6. Edit. The book has a new _____. [Edition]
7. Heal. The mother worried about her son’s ______. [Health]
8. Magic. The children watched the _____. [Magician]

### Both Shift Condition

1. Revise. This paper is his second _____. [Revision]
2. Produce. The play was a grand _____. [Production]
3. Explain. His excuse was a bad _______. [Explanation]
4. Absorb. She chose the sponge for its _____. [Absorption]
5. Permit. Father refused to give _____. [Permission]
6. Strong. She held on with all her _____. [Strength]
7. Extend. The student asked for an _____. [Extension]
8. Expand. The company planned an _____. [Expansion]
